# Technical essential aspects in robotic colorectal surgery: mastering the Da Vinci Si and Xi platforms

**DOI:** 10.1590/0100-6991e-20213007

**Published:** 2021-09-14

**Authors:** ANDRE LUIZ GIOIA MORRELL, ALEXANDER CHARLES MORRELL-JUNIOR, ALLAN GIOIA MORRELL, ELIAS COUTO ALMEIDA-FILHO, DUARTE MIGUEL FERREIRA RODRIGUES RIBEIRO, GLADIS MARIA PACILEO ANCHIETA RODRIGUES RIBEIRO, FRANCISCO TUSTUMI, JOSE MAURICIO FREITAS MENDES, ALEXANDER CHARLES MORRELL

**Affiliations:** 1 - Instituto Morrell, Cirurgia do Aparelho Digestivo Robótica e Minimamente Invasiva - São Paulo - SP - Brasil; 2 - Sociedade Beneficente Israelita Brasileira Albert Einstein, Cirurgia Geral e do Aparelho Digestivo Minimamente Invasiva e Robótica - São Paulo - SP - Brasil; 3 - Hospital Vila Nova Star, Cirurgia do Aparelho Digestivo Robótica e Minimamente Invasiva - São Paulo - SP - Brasil; 4 - Rede D’Or São Luiz, Cirurgia do Aparelho Digestivo Robótica e Minimamente Invasiva - São Paulo - SP - Brasil; 5 - Grupo Leforte, Cirurgia do Aparelho Digestivo, Bariátrica e Metabólica Robótica - São Paulo - SP - Brasil

**Keywords:** Robotic Surgical Procedures, Exoskeleton Device, Colorectal Surgery, Colorectal Neoplasms, Colonic Neoplasms, Procedimentos Cirúrgicos Robóticos, Exoesqueleto Energizado, Cirurgia Colorretal, Neoplasias Colorretais, Neoplasias do Colo, Procedimentos Cirúrgicos Minimamente Invasivos, Trato Gastrointestinal

## Abstract

**Background:**

laparoscopy surgery has many proven clinical advantages over conventional surgery and more recently, robotic surgery has been the emerging platform in the minimally invasive era. In the colorectal field, although overcoming limitations of standard laparoscopy, robotic surgery still faces challenging situations even by the most experienced colorectal surgeons. This study reports essentials technical aspects and comparison between Da Vincis Si and Xi platforms aiming to master and maximize efficiency whenever performing robotic colorectal surgery.

**Methods:**

this study overviews the most structured concepts and practical applications in robotic colorectal surgery in both Si and Xi Da Vinci platforms. Possible pitfalls are emphasized and step-wise approach is described from port placement and docking process to surgical technique. We also present data collected from a prospectively maintained database.

**Results:**

our early experience includes forty-four patients following a standardized total robotic left-colon and rectal resection. Guided information and practical applications for a safe and efficient robotic colorectal surgery are described. We also present illustrations and describe technical aspects of a standardized procedure.

**Conclusion:**

performing robotic colorectal surgery is feasible and safe in experienced surgeons hands. Although the Da Vinci Xi platform demonstrates greater versatility in a more user-friendly design with technological advances, the correct mastery of technology by the surgical team is an essential condition for its fully robotic execution in a single docking approach*.*

## RESUMO

**Introdução:**

a cirurgia laparoscópica demonstrou vantagens sobre a cirurgia convencional e, mais recentemente, a cirurgia robótica tem sido a plataforma emergente na era cirúrgica minimamente invasiva. Na prática colorretal, embora supere as limitações da laparoscopia, a cirurgia robótica ainda enfrenta situações desafiadoras, mesmo diante de cirurgiões colorretais experientes. Este estudo relata aspectos técnicos essenciais e comparação entre as plataformas Si e Xi Da Vinci com o objetivo de auxiliar e maximizar a eficiência na realização de cirurgia robótica colorretal regrada.

**Métodos:**

este estudo apresenta uma visão geral dos conceitos essenciais e aplicações práticas em cirurgia robótica colorretal nas plataformas Da Vinci Si e Xi. As potenciais dificuldades são enfatizadas e uma abordagem em etapas é descrita desde a colocação dos portais e seu processo de docking até a técnica cirúrgica. Também apresentamos brevemente dados coletados de um banco de dados mantido de forma prospectiva.

**Resultados:**

nossa experiência inicial inclui quarenta e quatro pacientes submetidos à cirurgia totalmente robótica padronizada na ressecção colônica e retal. Informações e aplicações práticas para uma cirurgia robótica colorretal segura e eficiente são descritas. Também são apresentadas ilustrações e dados breves da experiência.

**Conclusão:**

a cirurgia robótica colorretal é viável e segura nas mãos de cirurgiões experientes, no entanto ainda enfrenta desafios. Apesar da plataforma Da Vinci Xi demonstrar maior versatilidade em um design mais amigável com avanços tecnológicos, o correto domínio da tecnologia pela equipe cirúrgica é condição essencial para sua execução totalmente robótica em etapa única.

## INTRODUCTION

The benefits of laparoscopy for colorectal surgery have been demonstrated over the last years, showing non-inferiority oncological outcomes compared to conventional open surgery, whereas patient recovery and other short-term outcomes measures are improved. Although having a rapid progression of technology since its first attempt in 1991, inherent features of the laparoscopic procedures still represent difficulty even to highly experienced surgeons^1^. Limited range of motion of the rigid instruments, unstable image from a hand-held camera and limited visibility in the pelvis, particularly associated with rectal resections, are widespread technical challenges. 

Robotic surgery is an exciting emerging technique and has aimed to assist surgeons in overcoming some of these limitations. A high definition three-dimensional stereoscopic vision and magnification, improved ergonomics, wristed instruments and an intuitive stable and surgeon guided camera added to superior range of motion and freedom of movements are remarkable advantages^2^. The first worldwide disseminated da Vinci robotic system was initially introduced in 2006, with the Si platform (Intuitive Surgical Inc. Sunnyvale, CA, USA). Similarly, to initial laparoscopy, incorporation of robotic surgery demanded another surgeon’s learning curve and apprenticeship of technical aspects in a “new concept” of minimally invasive surgery. Operative room setup, patient positioning, type of docking and instruments retrieval were part of this learning and arguably slowed down its wholesale adoption. Potential benefits of the robotic approach with reduced conversion rates, shorter learning curve, reduced surgeon fatigue and better functional preservation when compared with conventional laparoscopy are discussed in colon and rectal surgery^3^. 

Nevertheless, there are still several limitations in robotic colorectal multiquadrant surgery experienced with the Da Vinci Si robotic system. The docking procedure isn’t simple and once the cart is docked, the position of the patient cannot be changed as needed without repeated undocking and redocking, prolonging operative time. Also, persistent arm clashing of the robot from one operative quadrant to another could result in difficulty in performing complete splenic flexure mobilization. In 2014, the new Da Vinci Xi robotic system was released and designed to address some of these limitations. In this study, we aimed to describe and guide technical aspects applied to a total robotic colorectal surgery, showing differences between both Si and Xi systems and assuring the maximum efficiency especially in left-sided colon and rectal surgery.

## METHODS

This is a guided review in a step-wise approach of robotic colorectal surgery and collects overviews of the most structured basic concepts in robotic surgery from a high-volume robotic surgery single group. A technical report with illustrations and practical applications in robotic colorectal surgery in both Si and Xi Da Vinci platforms is described. Possible pitfalls and standardized surgical steps are emphasized in a step-wise approach, from port placement and docking process to surgical technique, instruments, energy devices and anastomosis confection. Also, a description of a retrospective analysis in our group initial experience is reported. Patients undegoing a non-standardized totally robotic high or low anterior resection were excluded. 

### Da Vinci Si and Xi systems

Although both robotic platforms are put together as “robotic surgery”, the Si and Xi systems have their differences regarding its exoskeleton, instruments, ports and docking which are mandatory to be mastered before using them. The Si system patient’s exoskeleton is based in a large and vertical column, having its arms parallel to each other. Compared to the da vinci Xi, it has larger and more troublesome robotic arms to work, not rarely leading to external collisions. The robotic scope is a 12mm scope with a specific designed arm for its clutching, requiring a 12mm disposable trocar and not compatible with the 8mm ports for the robotic instruments. Overcoming some da Vinci Si limitations, the Xi latest model reinvented the concept of the patient cart design, with more versatility and flexibility^4^. The new exoskeleton is based in a boom-mounted architecture, with redesigned slim arms and patient clearance function providing greater range of motion and minimizing external collisions. Also, the robotic docking is possible from any angle and improves access around the patient at any quadrant. Compared to the Si bulky scope, the new designed 8mm robotic camera offers the surgeon a brighter surgical field with a higher resolution and longer scope. In addition, it requires no draping and can be attached to any of the four robotic arms, which lead to a more flexible and versatile vision of the operative field, as well as different options for port placements. All these features increase the flexibility and maneuverability of the Xi system and are expected to facilitate the performance of multiquadrant procedures.

### Operative Room setup and Patient preparation

The room setup may be similar to both da Vinci systems, however some key points must be highlighted. The robot is placed to the left of the patient, however, when using the Si platform, the docking is oblique, along an imaginary line between the anterior superior iliac spine and the umbilical scar; different from the Xi, whose docking is lateral. 

**Figure 1:**
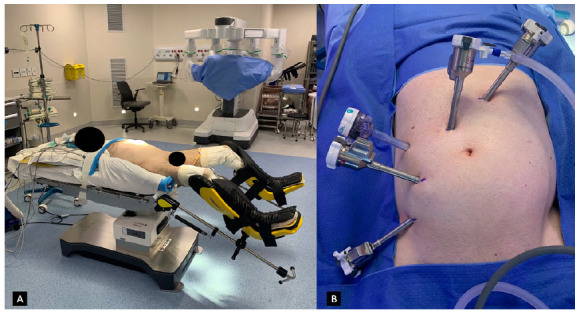
A. Patient positioning / B. Da Vinci Xi port placement.

The patient is placed in a modified lithotomy position and arms are close to the trunk to prevent injury and allow maximal space for the robot and the assistant (Figure 1A). Foley catheter insertion is mandatory and antibiotic prophylaxis is routinely achieved with administration of 1g intravenous ceftriaxone during anesthetic induction. Regardless of the platform, pneumoperitoneum is achieved by a Veress needle puncture in the umbilical site and carbon dioxide is insufflated until 15mmHg. After trocars insertions, the patient is placed in a 30° Trendelenburg and a tilt to the right side. The bedside assistant surgeon and scrub nurse remains on the right side of the patient to assist the console surgeon whereas the surgeon operates from the console.

### Port Placement and Docking

For port placement and docking, a single or double stage docking robotic technique for both Si and Xi is feasible. For the Si system, no standardized port placement was specifically designed by Intuitive Da Vinci for left colon and rectal surgery. Possible but not encouraged, our group decided not to perform the hybrid approach and discourse about its aspects in this study. Not irrelevant, we believe that whenever performing a standardized high or low anterior resection with this platform, a single or double stage technique should be programmed by the surgical team, because its decision could impact on reassignment, additional or better positioning of the trocars in the patient’s abdominal wall. Due to Si rigid robotic cart and inferior mobility of instruments range and higher incidence of arms collision, we encourage non-expert surgeons to perform a double stage docking robotic approach initially for a more ergonomic and efficient procedure. It’s important to state that ports may be shifted according to the habitus of the patient or the position of the internal anatomy, assuring a recommended da Vinci port distance ranging between 6-10cm. 

Despite being a platform, whose usage will be reduced and posteriorly be replaced by the latest Xi or X, we believe that learning in Si technology also is extremelly important. If a Si single docking approach is programmed, port placement is used having five working ports. This procedure is performed with the majority of its time in a two left hands setup; occasionally switching it to a two right hands scenario. A 12mm camera port is placed 2 to 3cm to the right of the umbilicus and slightly cranial to its imaginary line. Robotic port corresponding to arm one is placed in the right lower quadrant in the right iliac fossa, not too close to the right anterior superior iliac spine. Port of robotic arm two is placed in the epigastric area, at least 8cm from the camera and lefty to the midline and robotic port of arm three is placed in the left lower quadrant, lateral and anterior superior iliac spine, at the level of the umbilicus. This third robotic arm is reassigned during the procedure for a second left or second right hand instrument during the procedure according to the surgeon’s necessity, initially placed as a left hand. Lastly, an assistant port is placed in the right flank, ensuring the optimal ports triangulation for the bedside assistant. This assistant port is used for tissue manipulation and retraction, suction, irrigation and stapling if necessary. To avoid a port-in-port technique, the 5mm assistant port may be exchanged for a 12mm trocar. The procedure is performed using a monopolar scissors, a fenestrated bipolar forceps, a Cadiere or a Tip-up grasper and if necessary a Harmonic ACE Curved Shears. 

For a Si double stage approach, the procedure is divided into two steps, with the first colonic phase and splenic flexure mobilization; and the second, the pelvic phase. Da vinci patient cart should be initially docked more laterally to the patient and not oblique to minimize arms collision. An additional fourth robotic port is placed in the right side of the epigastric area, and the camera port may be positioned slightly more laterally to the single docking approach and assistant por can be slightly caudal. Also, the port of the arm three can be placed slightly below the level of the umbilical imaginary line. In the colonic phase, robotic arms are docked assuring the surgeon’s two right hands, by the port number one and three; and surgeons left hand with the robotic port number four. This can be achieved by having the Da Vinci three arm disposed together to the arm number one when draped or have its arm flipped and rotated by its robotic ‘elbow’. Once the colonic phase is accomplished, arms are undocked, patient cart is placed oblique and a standard two left hands docking setup is done, with the reassignment of the robotic third arm to the left side of the robotic cart, together to the arm number two; remaining the fourth robotic port undocked. 

The major difference between procedures using the Xi and the Si robotic systems is the arrangement of ports. For the Xi system, Intuitive Surgical provided a Universal Port Placement Guidelines, with a standard port placement for left lower abdominal procedures. Ports are recommended to be placed on a straight line 7cm apart configuration, distinct to the Si setup, whose ports are always encouraged to be in a non-linear pattern. For high and low anterior resections, the Intuitive’s port placement guidelines advises drawing a line from the right femoral head (lateral border of inguinal triangle) to where the left mid-clavicular line (MCL) crosses over the left subcostal margin. The camera port, planned to be docked in robotic arm two, is placed at the crossing of this imaginary line with the midline and subsequent ports one, three, and four are put at a distance of 8cm to other ports on this line. An assistant port is triangulated as far away as possible from da Vinci ports and lateral to the right MCL; between ports two and three. This port guideline orients a two right hands surgical procedure. Our group prefers to locate all trocars more laterally to the right side to enhance the workspace and facilitate the approach to the splenic flexure (Figure 1B). In this particular port placement, the camera port is placed laterally and superiorly to the umbilicus, not in its midline, and port one is placed slightly left to the midline. An assistant port is also placed in the right flank for the same purposes. This modified port setup allows a more lateral view of the structures and surgeons’ left hand may reach the splenic flexure more easily without arms collisions. 

**Figure 2:**
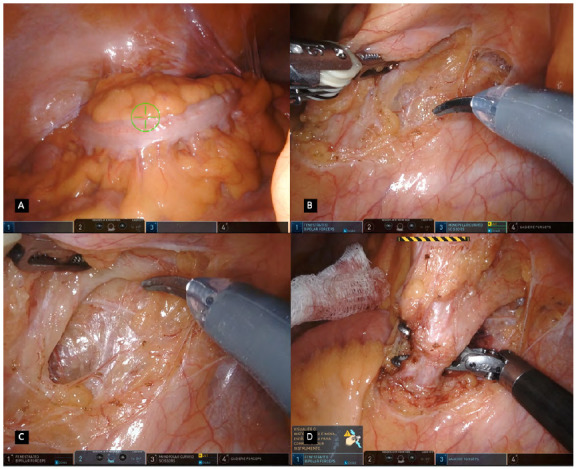
A. Robotic targeting / B. IMA pedicle and peritoneum incision / C. Preservation of hypogastric nerves and retroperitoneal fascia / D. Isolation of the inferior mesenteric artery at 1cm from its origin.

Once all ports are positioned, the patient is placed in the correct position and before docking commences, the omentum, transverse colon and small bowel are displaced cranially. The Xi robotic patient cart is docked in a lateral docking from the patient left side and the laser guided system displaying a green target is projected from the cart’s overhead boom aligned to the camera port corresponding to robotic arm two. The endoscope is then inserted into the cavity, pointed towards the sigmoid and inferior mesenteric artery projection and selected as the target anatomy; optimizing robotic arms configuration (Figure 2A). Adjustment of the Xi Flex joints on all arms to point towards the splenic flexure or pelvis is done aiming to open up space between arms to increase reach and avoid interference. The procedure is performed using the same instruments as the Si technology, with the exception of the possibility to use a robotic EndoWrist Stapler® attached to arm four and used to divide the rectum. If not available, similar to the Si system, a port-in-port technique in the arm four can be used for a disposable locking clips or laparoscopic stapling. 

Due to the identity of Xi robotic arms and possibility of instruments and the endoscope exchange between them, in case of surgeon’s preference, a different instrument disposal and docking is also possible for a two left-hands docking setup. The robotic camera should be placed in the arm three and arms one and two are assigned for the Cadiere grasper and fenestrated bipolar forceps respectively. 

This study has been approved by the ethics committee under number 40042420.2.0000.0087.

### Technical considerations

The procedures are quite alike between the two systems. The operation is performed in a medial to lateral approach having the inferior mesenteric artery (IMA) as the initial landmark. The IMA pedicle is tractioned and the peritoneum is incised separating the mesocolon from the retroperitoneal plane and primary vascular control is achieved by skeletonising and ligating the inferior mesenteric artery at 1cm from its origin in order to prevent injury to the hypogastric nerves (Figures 2 B,C,D). The dissection to assure the correct plane while preserving ureter and gonadal vessels requires most of the time a precise and tireless retraction. The ligation is done by applying disposable locking clips, with the assistant port or in a port-in-port technique; however, a vascular stapler can also be applied for its division. 

**Figure 3:**
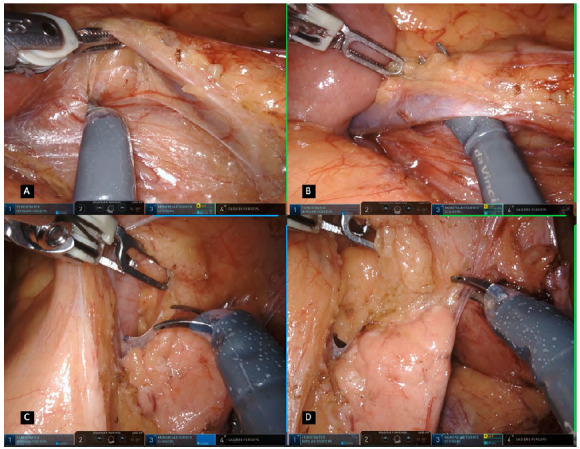
A. Dissection developing the plane between the mesocolon and Gerota’s fascia / B. Isolation and ligation of the inferior mesenteric vein / C. Access of the lesses sac after identification of the upper border of the pancreas and peritoneum incision / D. Dissection toward the tail of the pancreas for a splenic flexure mobilization.

**Figure 4:**
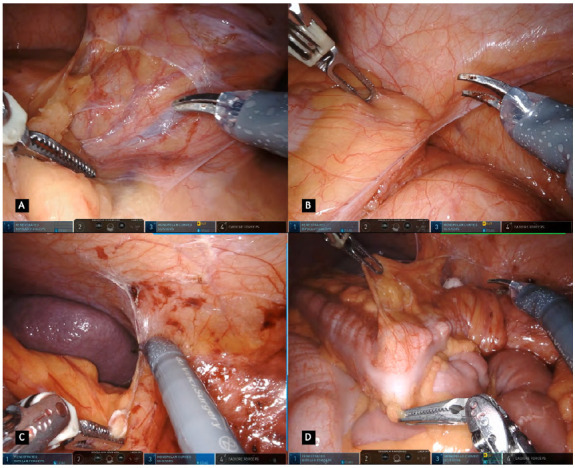
A. Initial lateral colonic mobilization by dividing the lateral peritoneal reflection / B. Bottom-up fashion colonic dissection along Toldt’ fascia / C. Division of the splenocolic ligaments and lateral attachments / D. Transverse colon retraction for gastrocolic ligament division.

The inferior mesenteric vein (IMV) is then pulled upwards and further dissection is achieved, developing the plane between the mesocolon and the Gerota’s fascia (Figure 3A). A sponge gauze is used to prevent mesocolon rupture during countertraction and the medial-to-lateral dissection is continued as far laterally as possible, with extreme caution not to cause injury to the IMV pedicle. The assistant surgeon plays an important role in providing additional countertraction during this part of the dissection. The duodeno-mesenteric fossa is explored and the IMV is dissected, ligated and divided at the level of the lower border of the pancreas (Figure 3B). The upper border of the pancreas is identified and the peritoneum covering its edge is cut to achieve the lesser sac in an avascular space exposing the posterior wall of the stomach (Figure 3C). Further dissection is performed cephalic and laterally toward the tail of the pancreas (Figure 3D). A gauze can be placed above the tail of the pancreas and other retroperitoneal structures used as landmarks for the dissection plane when completing the dissection along the Toldt’s line. The lateral colonic mobilization is commenced by dividing the lateral peritoneal reflection, commonly performed in a bottom-up fashion along the Toldt’ fascia with great care not to damage the retroperitoneal structures (Figures 4A, B). If a complete splenic flexure mobilization is needed, at this point, division of the gastrocolic and the splenocolic ligaments is performed, followed by lateral colonic mobilization towards the spleen (Figures 4C, D). If necessary, in high BMI, tall or male patients, a reverse Trendelenburg position through manual adjustment or integrated table motion feature can help in this final step of the splenic flexure mobilization to displace the transverse colon downwards. 

**Figure 5:**
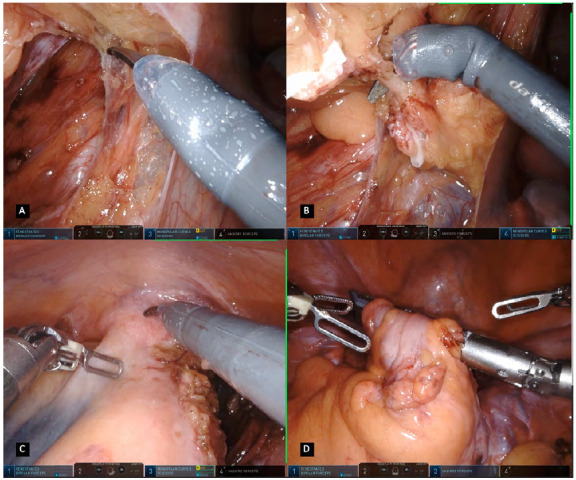
A. Rectal dissection in the posterior avascular cleavage plane between the visceral and the parietal fascial sheets / B. Rectal artery isolated whenever not performing a total mesolectal excision / C. Rectum retraction and anterior dissection using monopolar scissors / D. Rectal division using a laparoscopic or robotic.

In the pelvic phase, the dissection is performed according to the principles of the avascular cleavage plane between the visceral and the parietal fascial sheets, allowing maximal protection of the hypogastric nerves and the inferior hypogastric plexus (Figure 5A). For better exposure, in female patients, a straight needle or transvaginal uterus manipulator may be used to retract the uterus up to the anterior abdominal wall. The rectum is pulled and dissection commences posteriorly (Figure 5B). This plane consists of fine areolar tissue that can be easily divided with the monopolar scissors all the way to the level of the anococcygeal ligament. The Cadiere forceps are used to lift and retract the rectum outside the pelvis while the surgeon’s left hand is used for countertraction. Anterior and lateral attachments are further dissected, and if an extremely low rectal mobilization is required (Figure 5C), the 30° scope is turned looking upwards to provide a better view of the pelvic floor and rectal distal margin. Rectal stapling is achieved after a complete exposure of its wall in cases not requiring a total mesorectal excision (TME). The rectum is divided using a laparoscopic stapler through either the bed-side assistant port or the left iliac fossa port site in a port-in-port technique (Figure 5D). A 60mm stapler can be used when performing a high transection of the rectum or in patients with wider and large pelvis. When aiming a low rectal transection, a 45mm stapler fits more easily in the confines of a narrow pelvis, and stapling can be performed in a vertical anterior to posterior orientation if necessary. If available, a robotic EndoWrist Stapler® can be attached to arm four and used to divide the rectum, in a 12mm robotic port. 

**Figure 6:**
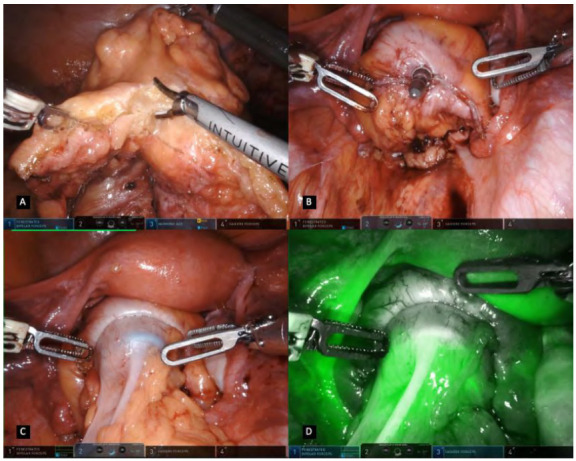
A. Colonic proximal margin settled and mesocolon division for specimen extraction / B. Rectal stump with a circular stapler prepared for a colorectal anastomosis / C. Conventional filter visualizing the colorectal anastomosis / D. Intravenous ICG and Firefly filter used accessing anastomosis integrity with real-time fluorescence guided image.

Following its division, the colonic proximal margin is settled, the mesocolon is divided according to its direction and the specimen is extracted through a 4cm transumbilical laparotomy incision using a wound protector (Figure 6A). The robotic system is undocked and the patient cart is withdrawn so the surgeon can have complete access to the operative field. The anvil is positioned in the proximal bowel with a purse string suture and placed into cavity again followed by reestablishment of the pneumoperitoneum and robotic docking. A standard tension-free and hermetic colorectal anastomosis is performed with the circular stapling device (Figures 6B, C). Routinely, intravenous indocyanine green (ICG) is used to access anastomosis integrity with real-time fluorescence guided image (Figure 6D). A bolus of intravenously 7.5mg ICG is administered according to the surgeons’ command followed by a 10mL saline bolus and activation of Firefly™ technology filter. Finally, the anastomosis is routinely tested with an air leak test and if positive, additional stitches are performed in the leak area. A silicon drain is inserted into the pelvis and withdrawn through the right iliac fossa port. 

Some technical variations are also possible concerning specimen extraction and performing the colorectal anastomosis previously described by Morrell et al.^5^, not particularly related to this study. The specimen withdrawal and the anvil placement can be achieved through natural orifices; although, as highlighted they are not encouraged in oncological cases. Two types of anastomosis can be performed easily due to the anvil’s positioning, in an end-to-end or lateral-to-end fashion, depending on the surgeons’ choice. Not only the circular stapled but also hand-sewn colorectal anastomosis is possible utilizing a robotic needle driver and 3-0 barbed suture.

## RESULTS

Our early experience includes a total of forty-four patients who underwent this reported standardized robotic high or low anterior resections, for benign and malignant diseases. Procedures were performed utilizing both Si and Xi platforms, with single and double stage approaches in the first, and single docking technique in the latter. No conversion to laparoscopic or open surgery nor intraoperative complications were seen. Patients postoperative period was uneventful and no anastomotic leak or further complications have been documented. During the initial experience, over thirty colorectal high and low anterior procedures were performed without a systematic approach and different technical aspects. Pericolonic resections are not described or even counted in this group experience or learning curve as this technical approach doesn’t fit in the described standardized purpose. This group of sequential operations was fundamental to draw a learning curve and show possible pitfalls when obtaining competency. Patient and ports positioning, robotic docking and technical steps were improved during the experience. Learning curves for robotic surgery may commonly be divided into three performance phases and its aspects however are not the goal of this report. Therefore, any previous non-standardized robotic left-sided colorectal procedures non-accounted for this group.

## DISCUSSION

Although minimal access surgery has become the goal standard for colorectal surgery, laparoscopy surgery remains limited in some cases with inherent difficulties. Robotic approach has proven to overcome some of its limitations with a stable operating camera, fine motion scaling, three-dimensional imaging, articulated instruments and superior dexterity^6^. In the rectal procedures, the utilization of robotic technology makes even more sense within the narrow confines of the pelvis, where visibility is usually limited and maneuverability of rigid laparoscopic instrumentation is poor. 

Recently, more and more colorectal cancer patients have been operated robotically, and early experiences with different robotically assisted colorectal procedures have been described in the literature^7^ ^,^ ^8^. Although some studies including a meta-analysis of randomized controlled trials and propensity-score-matched cohorts have shown reduced rates of conversion to open procedures, robotic assisted surgery also faces some technical challenges^9^. Intrinsic to colorectal operations, a multi-quadrant abdominal dissection may be necessary depending on the procedure, more particularly for left-sided colon or rectal cancer; with partial or complete takedown of the colonic splenic flexure^10^. The da Vinci Si platform, however, is basically not designed for multi-quadrant abdominal dissection and possibly slowed down its wholesale adoption in the colorectal field compared to other surgical specialties. Currently, the issues confronting robotic assisted procedures are especially related to the operative times and costs, and particularly left-colon and rectal surgery related, the Si system complex docking process and persistent arm clashing play an important role. Therefore, some surgeons have even developed a double-docking technique to take down the colonic splenic flexure robotically^11^, as well as others have reported a robotic hybrid technique, in which the rectal phase was resected robotically and the colonic splenic flexure was mobilized laparoscopically.

The Da Vinci Xi® multiport surgical platform developed by Intuitive Surgical was released in 2014 and came up with several technological advances to address previous Si platform issues. The evolution of the robotic platforms towards improved usability introduced the Xi, a fourth-generation robot, promising an easier docking, improved ergonomics, wider range of motion and particularly important, a system that allows a multiquadrant surgery. The improvements in design are notorious. The boom-mounted architecture, with narrower arms and new clutch features allows a much more user-friendly docking. With a targeting device, additional flex joints, longer instruments with greater reach and a slimmer endoscope that can move between ports; colorectal surgery was hypothesized to have greater efficiency. The new EndoWrist Stapler has greater movement with a 120-degree articulation wrist allied to the SmartClamp technology, permitting optimal rectal transection with adequate tissue compression and three-dimensional vision. Therefore, early literature reported initial experience showing surgical feasibility in a single docking and single-phase technique using the new Xi technology^12^. Also, Protyniak et al.^13^ reported a higher rate of splenic flexure mobilization during colorectal surgery using the Xi robotic system in comparison with the Si system. 

In the Brazilian scenario, the introduction of robotic surgery still faces huge difficulties. We believe that training robotic surgeons is a highly demanding and expensive task, requiring experienced surgical staff besides the robotic platform and its instrumentals. A detailed robotic approach showing its most important pitfalls and technical considerations could facilitate training, decrease surgeons’ learning curve and enable reproducibility. During our group learning curve, a non-negligible amount of port placement and docking difficulties occurred resulting in apprenticeship for further cases. In this study, we aimed to testify and clarify both Si and Xi robotic generations characteristics, showing the imperative anatomical concepts and surgical steps of high and low anterior resections by optimizing the configuration of port placement and docking strategy. Despite the intrinsic limitations of this study, trained surgical groups familiarized with the robotic platform and understanding better the robotic system could result in a more effective procedure. As surgeons increase their armamentarium and robotic skills, more complex and challenging operations would be eligible for this minimally invasive approach.

## CONCLUSION

Robotic surgery is an emerging technique and provides a precise and more ergonomic dissection due to three-dimensional stereoscopic vision and articulated instruments. To maximize success on robotic high or low anterior resection, mastering the platform and its concepts is mandatory. The robotic colorectal approach requires appropriate port placement and optimal docking angle to minimize intraoperative difficulties. Familiarity with the operative characteristics and full use of its technology functions may improve the performance of the robotic surgeon. This study reports our group initial experience and masters both Si and Xi robotic technical considerations in colorectal surgery.
